# Metric magnetic resonance imaging analysis reveals pronounced substantia-innominata atrophy in dementia with Lewy bodies with a psychiatric onset

**DOI:** 10.3389/fnagi.2022.815813

**Published:** 2022-10-05

**Authors:** Niels Hansen, Sebastian Johannes Müller, Eya Khadhraoui, Christian Heiner Riedel, Philip Langer, Jens Wiltfang, Charles-Arnold Timäus, Caroline Bouter, Marielle Ernst, Claudia Lange

**Affiliations:** ^1^Department of Psychiatry and Psychotherapy, University Medical Center Göttingen, Göttingen, Germany; ^2^Institute of Diagnostic and Interventional Neuroradiology, University Medical Center Göttingen, Göttingen, Germany; ^3^German Center for Neurodegenerative Diseases (DZNE), Göttingen, Germany; ^4^Neurosciences and Signaling Group, Department of Medical Sciences, Institute of Biomedicine (iBiMED), University of Aveiro, Aveiro, Portugal; ^5^Department of Nuclear Medicine, University Medical Center Göttingen (UMG), Georg August University, Göttingen, Germany

**Keywords:** Lewy body dementia, MRI, atrophy, psychiatry, substantia innominata

## Abstract

**Background:**

Dementia with Lewy bodies (DLB) is a type of dementia often diagnosed in older patients. Since its initial symptoms range from delirium to psychiatric and cognitive symptoms, the diagnosis is often delayed.

**Objectives:**

In our study, we evaluated the magnetic resonance imaging (MRI) of patients suffering from DLB in correlation with their initial symptoms taking a new pragmatic approach entailing manual measurements in addition to an automated volumetric analysis of MRI.

**Methods:**

A total of 63 patients with diagnosed DLB and valid 3D data sets were retrospectively and blinded evaluated. We assessed atrophy patterns (1) manually for the substantia innominata and (2) *via* FastSurfer for the most common supratentorial regions. Initial symptoms were categorized by (1) mild cognitive impairment (MCI), (2) psychiatric episodes, and (3) delirium.

**Results:**

Manual metric MRI measurements revealed moderate, but significant substantia-innominata (SI) atrophy in patients with a psychiatric onset. FastSurfer analysis revealed no regional volumetric differences between groups.

**Conclusion:**

The SI in patients with DLB and a psychiatric-onset is more atrophied than that in patients with initial MCI. Our results suggest potential differences in SI between DLB subtypes at the prodromal stage, which are useful when taking a differential-diagnostic approach. This finding should be confirmed in larger patient cohorts.

## Introduction

Dementia with Lewy bodies (DLB) is the second most frequent neurodegenerative dementia (Walker et al., 2015). It is presumed that DLB is frequently misdiagnosed as core symptoms might be missing in particular in early stages or symptoms might coincide with Alzheimer’s or Parkinson’s disease (Rizzo et al., 2018). Recently novel research criteria (McKeith et al., 2020) have been defined that categorize the onset in patients with Lewy bodies in a prodromal stage involving three groups: (1) mild cognitive impairment (MCI)-onset, (2) psychiatric-onset, and (3) delirium-onset. The psychiatric symptoms at disease onset are reported to include a psychiatric symptomatic spectrum of depression, anxiety or delusions (McKeith et al., 2020). Our aim is to investigate differences in magnetic resonance imaging (MRI) volumetry between the three onset-types in patients with DLB. The MRI morphology of DLB is diverse and difficult to grasp. Investigations from Hanyu et al., 2002, 2005, 2007 described atrophy of the substantia innominata (SI) in respect to DLB. The SI is a narrow area in the basal forebrain located below the globus pallidus on a level with the anterior commissure inclusive of the nucleus basalis of Meynert. The mean SI volume is known to be reduced in patients with DLB and Parkinson’s dementia (PDD) than in those with Alzheimer’s dementia (AD) (Kim et al., 2011) supporting the SI’s potential important role in the pathogenesis of alpha-synucleinopathies. In our study we therefore took a new, pragmatic approach to measure the SI in DLB patients presenting the main subtypes, namely psychiatric- and MCI-onset. We also carried out automated segmentation and volumetric measurements. Finally, we compared the two methods in patients with DLB separated by their initial symptoms: MCI versus a psychiatric episode (PSY).

## Materials and methods

### Patients

In our retrospective single-center, observational study, we enrolled patients with DLB in their dementia stage assessed according to the latest international DLB consensus criteria (McKeith et al., 2017) as the first step. They are the same patient population as reported on in a recent publication (Khadhraoui et al., 2022). Partial evaluations of the patient cohort that do not focus on this study’s research question have been published (Khadhraoui et al., 2022). In the second step we reclassified the patients with a final DLB diagnosis according to their symptoms’ onset as those with an MCI-onset, those with an onset of psychiatric symptoms, and patients with a delirium onset according to the novel prodromal-DLB criteria according to McKeith et al. (2020). An MRI of the brain with a 3D T1 data set was used in all patients independent of the MRI manufacturer. The field strength was not considered in the analysis. We took manual measurements and calculated volumetry using 63 Sequences (50 1.5-Tesla, 13 3-Tesla, 46 T1 MPRAGE, 17 T1 VIBE). For the subgroup with the MCI-onset, we evaluated 30 sequences (23 1.5-Tesla, 18 T1 MPRAGE). 30 sequences were also evaluated in patients with a psychiatric onset, (26 1.5-Tesla 26, 25 T1 MPRAGE). Ethics approval was obtained from the ethics committee of the University Medical Center Göttingen. A study protocol is provided in the supplemental data. This retrospective study adhered to the 2013 Helsinki Declaration.

### Diagnostic and clinical examination

We classified patients in line with the latest consensus criteria (McKeith et al., 2017). Our patients were recruited from the Picture Archiving and Communication System (PACS) database system and their medical records were examined. β-amyloid 42 (Aβ42) and β-amyloid 40 (Aβ40) from cerebrospinal fluid (CSF) were determined in the Neurochemistry Laboratory of the Neurology Department, University Medical Center Göttingen using commercially available INNOTEST^®^ β-AMYLOID (1–42) ELISA kit (Fujirebio) and ELISA from IBL [AMYLOID BETA (1–40)]. The ratio Aß42/40 was considered as pathological when values are < 0.5. We relied on laboratory internal reference values for this cut-off value. The cut-off level results from multiplying the ratio Aβ42/40 by the factor 10.

### Magnetic resonance imaging analysis

Magnetic resonance imaging 3 D T1 data were acquired from two distinct MRI scanners (1.5 Tesla Siemens AvantoFit and 3.0 Tesla Siemens Magnetom/PrismaFit) between 2013 and 2020. Two independent raters evaluated the patients with DLB and our control cohort. The raters, who were blinded regarding the patient data including DLB diagnosis, are radiologists with (1) 4 years (rater 1, EK) and (2) 8 years (rater 2, ME) of neuroradiologic experience in neuroimaging dementia *via* MRI. All subjects were scanned in sagittal orientation with a voxel resolution of 1.0 mm × 1.0 mm × 1.0 mm with the following parameters: MPRAGE: 1.5T (Siemens Avanto fit): TR 1.700 ms, TE 2.46 ms, flip angle 8°, and TI 900 ms. 3.0T (Siemens Prisma fit): TR 2.000 ms, TE 2.98 ms, flip angle 9°, and TI 900 ms. VIBE 3D iso: 1.5T (Siemens Avanto fit): TR 5.770 ms, TE 2.38 ms, flip angle 10°. 3.0T (Siemens Prisma fit): TR 4.960 0 ms, TE 2.24 ms, flip angle 9°. We also applied a visual analogue score assessing the SI’s cortical thickness using a 4-step scale: 0 (no atrophy), 1 (mild atrophy), 2 (moderate atrophy), 3 (severe atrophy), as described in Khadhraoui et al. (2022).

#### Metric magnetic resonance imaging analysis

**FIGURE 1:**
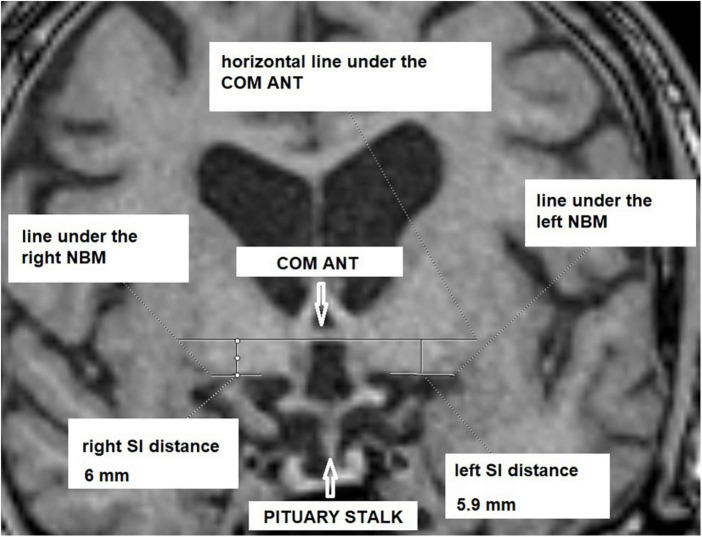
Measurement example. COM ANT, commissura anterior; SI, substantia innominata; NBM, nucleus basalis of Meynert.

Two neuroradiologists (ME and EK) took independent metric measurements. These neuroradiologists were blinded to patient data and diagnosis. A coronal image was reconstructed utilizing each patient’s the 3D T1 data set. An algorithm was used to assess images that focuses on the middle of the pituitary stalk and anterior commissure (Figure 1): (1) A horizontal line was drawn under the anterior commissure, (2) two horizontal lines under the nucleus basalis of each hemisphere were analyzed, (3) two vertical lines from the anterior commissure line through the middle of each of the nucleus basalis lines were considered and (4) the distances were assessed. The images were reconstructed as described in Khadhraoui et al. (2022) and saved in our Picture Archiving and Communication System (PACS systems). Measurements were taken using these reconstructed images with excellent interrater reliability.

#### Volumetric magnetic resonance imaging analysis

The 3D Slicer (Version 4.10.2^1^) was utilized for transforming from DICOM (Digital Imaging and Communications in Medicine) to NIFTI (Neuroimaging Informatics Technology Initiative) file format. Segmentation was done using Fastsurfer (Henschel et al., 2020), Version commit dabf1e02e6253cac8bd3d641958b01e5348ea0e7^2^ with the procedure call: run_fastsurfer.sh -fs_license $FREESURFER_HOME/license.txt -sd $out_path -sid $filename -t1 $f/$filename.nii -parallel -threads 24 -batch 64 -order 3 -vol_segstats. Surface statistica were achieved using FMRIB Software Library v6.0 (FSL 6.0), Version 6.0.4.^3^ Used graphic card was GPU nVidia GV100, Driver 455.45.01, CUDA Version 11.1. Ubuntu 18.04.5 LTS was used as operating system. Each patient’s data from the stats folder was stored in a separate Excel-file. Fast surfer’s standard segmentation algorithm (Fischl, 2012), and Desikan-Killiany-Tourville DKTatlas.aseg.stats (Potvin et al., 2017; Mikhael and Pernet, 2019; Yaakub et al., 2020) were used for segmentation and volumetric analysis. Each patient’s segmentation was manually controlled.

### Statistical analysis

The Statistica, version 13 program (TIBCO Software Inc., Palo Alto, CA, USA) was utilized for statistical analyses. The data were tested for normal distribution *via* Shapiro–Wilk-Test. Student’s *t*-tests were performed for analysis of groups (PSY, MCI) with or without amyloidopathy. A result was considered as significant if *P* < 0.05. Interrater agreement was analyzed utilizing intraclass correlation coefficient (ICC). The ICC was calculated exploiting the libraries in R Version 4: irr, readxl, lpSolve, and psych. We performed corrections for multiple comparisons using linear models with covariates using lm () in r. We also ran a correction for multiple comparisons *via* the Bonferroni–Holm method.

## Results

### Participants

Dementia with Lewy bodies diagnoses according to the current McKeith criteria (2017) were made in the cohort of 63 patients (27 females) with DLB from psychiatric and neurophysiological files with available MRIs including a 3D T1 sequence (Table 1 for demographic and clinical data). Mean age (at time of MRI) of the cohort was 74.9 ± 7.0 years (range 53–89 years). Initially, 30 patients (11 females) suffered from (i) MCI, 30 patients (15 females) from (ii) psychiatric symptoms (PSY), and in three (1 female), the diagnoses were caused (iii) by delirium. The MCI group’s average age was 74.7 ± 6.6 years, the PSY group’s 76.0 ± 7.4 years, and the delirium group’s (iii) 70.8 ± 5.9 years. The age of the subgroups did not differ significantly (Table 1), nor did their MMSE scores, education levels, and disease duration from initial presentation to dementia diagnosis (Table 1).

**TABLE 1:** Demographic and clinical data of patients.

Parameter	DLB, *n* = 63	DLB with psychiatric-onset, *n* = 30	DLB with MCI-onset, *n* = 30	DLB with delirium-onset, *n* = 3
Age	74.9 ± 7.4, *n* = 63	76.0 ± 7.4, *n* = 30	74.7 ± 6.6, *n* = 30	70.8 ± 5.9, *n* = 3
Disease duration	2.8 ± 2.4, *n* = 23	3.6 ± 2.8, *n* = 13	1.9 ± 1.4, *n* = 9	0.5, *n* = 1
Education	21 ± 0.8, *n* = 21	9 ± 0.7, *n* = 9	12 ± 0.9, *n* = 12	–
MMSE	21.1 ± 6.4, *n* = 40	22.0 ± 6.2, *n* = 23	20.8 ± 6.6, *n* = 23	14 ± 1.4, *n* = 2
**CSF data**				
T-tau	399 ± 219, *n* = 55	379 ± 241, *n* = 24	416 ± 241, *n* = 27	170 ± 134, *n* = 2
Ptau181	60 ± 26, *n* = 55	58 ± 19, *n* = 24	64 ± 30, *n* = 27	27 ± 17, *n* = 2
Aß42	875 ± 342, *n* = 55	888 ± 309, *n* = 24	883 ± 388, *n* = 27	643 ± 135, *n* = 2
Ratio Aß42/40	0.9 ± 0.4, *n* = 55	0.8 ± 0.3, *n* = 24	0.89 ± 0.46, *n* = 27	1.12 ± 0.68, *n* = 2

Aβ42, β-amyloid 42; CSF, Cerebrospinal Fluid; MMSE, Mini Mental Status Examination; n, number; ptau181, phosphorylated tau protein 181; Ratio Aβ42/40, ratio β-amyloid 42/40; T-tau, T-tau protein; DLB, dementia with Lewy bodies; MCI, mild cognitive impairment.

### Manual measurement

The mean measured SI-distances in all patients were 6.3 ± 1.2 mm (mean ± standard deviation) for the left and 5.9 ± 0.9 mm for the right hemisphere.

### Automated volumetric analysis

Details on volumetric analyses are in Supplementary Table 1A.

### Different atrophy patterns by clinical group/initial symptoms

Manual measurements of the left SI (*p* < 0.02) showed significantly more atrophy in patients with psychiatric symptoms than in patients with MCI, as shown in Table 2. A multi parametric *t*-test analysis showed significant group differences (MCI > PSY) in only three of 120 volumetric parameters: the left (*p* < 0.03) and right (*p* < 0.02) paracentral cortices and left pars orbitalis cortex (*p* < 0.05), as show in Table 1. After correction for multiple comparisons *via* Bonferroni-Holm, no significant volumetric parameters remained. More T1 MP-RAGEs than VIBEs were done in patients with initial psychiatric symptoms (*n* = 25) than in patients with MCI (*n* = 18), attributable to the in-hospital MRI protocol. This led to an overestimation of cortex volumes in the MCI patients, thus explaining the deviation between the left and right paracentral cortices and left pars orbitalis: in a separate evaluation, these volumes were sequence-dependent, and disappeared in a separate MPRAGE only analyses, as shown in Supplementary Table 1B, but the differences in the manual measurements remain. The mean sums of all cortex volumes were 331000 ± 61023 mm^3^ for T1 VIBE sequences, and 366000 ± 43038 mm^3^ for T1 MPRAGE.

**TABLE 2:** Short overview of volumetric analysis.

Parameter	MCI		PSY		*P*-value
		
	Mean	SD	Mean	SD	
T1 MPRAGE (1-yes, 0-no)	0.6	0.50	0.8	0.38	0.046
Manual distance SI left (mm)	0.67	0.13	0.60	0.11	0.018
Manual distance SI right (mm)	0.58	0.09	0.59	0.09	0.701
ctx-lh-parahippocampal (mm^3^)	1592.6	395.53	1696.9	342.58	0.279
ctx-lh-paracentral (mm^3^)	2983.5	576.37	3362.7	671.75	0.022
ctx-lh-parsopercularis (mm^3^)	3130.6	698.78	3048.5	577.68	0.621
ctx-lh-parsorbitalis (mm^3^)	1394.2	390.48	1586.8	348.13	0.048
ctx-lh-parstriangularis (mm^3^)	3281.0	711.30	3164.0	641.13	0.506
ctx-lh-pericalcarine (mm^3^)	1419.4	362.91	1322.7	346.45	0.296
ctx-rh-parahippocampal (mm^3^)	1492.4	356.94	1580.0	281.22	0.296
ctx-rh-paracentral (mm^3^)	2916.6	563.54	3309.0	644.65	0.015
ctx-rh-parsopercularis (mm^3^)	3035.6	671.51	2952.9	599.12	0.617
ctx-rh-parsorbitalis (mm^3^)	1564.2	357.33	1594.8	318.48	0.727
ctx-rh-parstriangularis (mm^3^)	3057.2	782.22	2795.4	657.78	0.166
ctx-rh-pericalcarine (mm^3^)	1580.6	381.00	1483.4	342.86	0.303

ctx, cortex; lh, left hemisphere; rh, right hemisphere; MCI, mild cognitive impairment onset; PSY, psychiatric onset; SD, standard deviation.

### Positive β-amyloid markers and substantia-innominata atrophy

**FIGURE 2:**
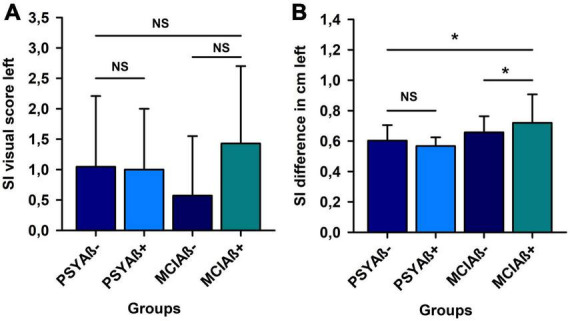
β-amyloid pathology and substantia innominata atrophy. Four different groups regarding their β-amyloid status were compared [PSY with Aβ42/40 ratio < 0.5 (PSYAβ+, *n* = 3) and PSY with Aβ42/40 ratio > 0.5 (PSYAβ-, *n* = 21), MCI with Aß42/40 ratio < 0.5 (MCIAβ+, *n* = 7) and MCI with Aβ42/40 ratio > 0.5 (MCIAβ-, *n* = 21)]. The visual score on the left **(A)** SI did not differ between groups. However, the left SI difference (in mm) was significantly less in the PSYAβ- than the MCIAβ+ group **(B)**. SI, substantia innominata. *Means *p* < 0.05, NS means non.-significant. Aß42/40 ratio, ratio of amyloid-ß42/amyloid-ß40; PSY, psychiatric-onset; MCI, psychiatric onset.

We formed four groups according to their β-amyloid marker’s positivity [PSY with Aβ42/40 ratio < 0.5 (PSYAβ+, *n* = 3) and PSY with Aβ42/40 ratio > 0.5 (PSYAβ-, *n* = 21), MCI with Aβ42/40 ratio < 0.5 (MCIAβ+, *n* = 7) and MCI with Aβ42/40 ratio > 0.5 (MCIAβ-, *n* = 21)]. The visual score on the left (Figure 2A) and right SI (data not shown) did not differ between groups (PSYAβ+ vs. PSYAβ-, MCIAβ+ vs. MCI Aβ-, PSYAβ- vs. MCIAβ-, PSYAβ+ vs. MCIAβ+, PSYAβ-vs. MCIAβ+, PSYAβ+ vs. MCIAβ-). Furthermore, when we measured the difference in the right SI (in mm) between groups, no differences appeared (PSYAβ+ vs. PSYAβ-, MCIAβ+ vs. MCI Aβ-, PSYAβ- vs. MCIAβ-, PSYAβ+ vs. MCIAβ+, PSYAβ-vs. MCIAβ+, PSYAβ+ vs. MCIAβ-) (data not shown). However, the left SI difference (in mm) was significantly less in the PSYAβ- than the MCIAβ+ group (Figure 2B).

### Interrater agreement

The single score ICC (A, 1) was 0.889 with a 95%-Confidence Interval of 0.82–0.93.

## Discussion

In our study of 63 patients with confirmed DLB and a 3D T1 dataset, we demonstrate for the first time a smaller SI *via* manual MRI thickness analysis in DLB patients with a psychiatric-onset than in DLB patients with an MCI-onset. The pronounced atrophy of the SI in DLB patients with a psychiatric-onset is surprising, as a recent study demonstrated that the nucleus basalis of Meynert is as well atrophied in MRI in prodromal DLB patients with an MCI-onset (Schumacher et al., 2021). The nucleus basalis of Meynert is a cholinergic part of the SI (Liu et al., 2015). This evidence indicates that in both a psychiatric- and MCI-onset of DLB, the SI is atrophied, but also that the atrophy in patients with a psychiatric-SI is linked to alpha-synucleinopathy in DLB, but less so in AD (Kim et al., 2011). We noticed less atrophy in MCI patients presenting positive β-amyloid markers than in those patients with a psychiatric-onset with no hints of amyloidopathy suggesting that positive ß-amyloid markers are an irrelevant factor concerning the observed SI’s atrophy pattern in patients with a psychiatric-onset. This assumption is further corroborated by the fact that the SI is not apparent between groups if we compare those psychiatric-onset and MCI-onset patients who share positive β-amyloid markers. The alpha-synucleinopathy suspected to affect the SI is very likely to play a major role in those DLB patients suffering from a psychiatric-onset. However, this finding is controversial and may dependent on the MRI evaluation algorithm, as another study demonstrated more prominent atrophy of the SI in AD than in DLB patients in voxel-based morphometry in MRI (Whitwell et al., 2007). Nevertheless, the SI atrophy was revealed in our study *via* a manual metric approach, revealing that methodological factors might explain such differences. Furthermore, our cohort differs somewhat to cohorts with neurological patients as we investigated a neuropsychiatric cohort of DLB patients. Parts of the SI like the nucleus basalis Meynert play a relevant role in cortical connectivity regarding memory, motor and visual functions, as a recent study by Oswal demonstrated (Oswal et al., 2021) indicating potential causes generating psychiatric symptoms in DLB patients with atrophied SI. The SI’s function including the nucleus basalis of Meynert is still enigmatic; animal and human studies have depicted its role in behavioral dysfunction such as aggressive behavior (Zhu et al., 2021), impaired attention and memory (Eck et al., 2020; Nemy et al., 2020), perception involving visual discrimination (Evenden et al., 1989) and the processing of novelty and reward (Wilson and Rolls, 1990; Wright et al., 2003; Martinez-Rubio et al., 2018), as well as aversive signal processing through a SI-amygdala connection (Cui et al., 2017). The aforementioned SI functions imply that such atrophy might trigger its malfunctioning, thereby probably explaining psychiatric symptoms occurring at DLB patients’ disease onset such as mood and perceptual dysfunction as well as anxiety.

The measurement method applied in our study is a pragmatic, sequence-independent approach to assess atrophy of the SI substance that helped us demonstrate differences in DLB subgroups. More precisely, it revealed more severe atrophy of the left SI in patients with psychiatric-onset compared to those with an MCI-onset.

In contrast to this observation, we detected no significant differences in the automatically calculated volumes in both groups using FastSurfer and DKTAtlas. It seems that the SI is either too small or insufficiently mapped by existing volumetric atlases. It is not yet possible to automatically detect SI atrophy *via* FastSurfer.

### Limitations

Volumetric measurements in MRI remain difficult despite simplified and standardized automated volumetry analyses. Our evaluation identified sequence-dependent differences that fail to facilitate volumetric analyses. In addition, the same sequences were not always done on the same scanner. As our study was retrospective, this issue could not be optimized. The additional volumetric analysis in patients exhibiting β-positive amyloid markers reveal a tendency that seems to indicate that amyloidopathy is not the main factor in SI atrophy in patients with a psychiatric-onset, although our patient numbers are too small to draw clear conclusions from these results. Furthermore, standard segmentation of the frontobasal brain is not very accurate. Thus we recommend for a future study with a larger sample to apply extended standard volumetry. Such segmentation should be carefully evaluated to see whether they are useful.

## Conclusion

The SI in patients with DLB who suffer a psychiatric-onset is more atrophied than it is in patients whose DLB onset is characterized by MCI. Our work shows that the latest basic volumetric segmentation algorithms do not detect every kind of atrophy. The algorithms are significantly limited in identifying atrophied areas despite being so easy to use. The diagnosis of atrophy in the SI in DLB patients with a psychiatric onset supports the hypothesis of a relevant pathogenic role of the SI in DLB’s pathogenesis characterized by a psychiatric-onset.

## Data availability statement

The raw data supporting the conclusions of this article will be made available by the authors, without undue reservation.

## Ethics statement

The study involving human participants was reviewed and approved by the Ethics Committee University Medical Center Göttingen. Written informed consent for participation was not required for this study in accordance with the national legislation and the institutional requirements.

## Author contributions

EK and ME evaluated the manual measurements. SM and CL were the project administrators and organized the data curation, measurements, and design. CL, NH, and SM did the literature review and wrote the manuscript. PL administrated the FastSurfer, made the volumetric measurements, and supports statistical analysis. CB, EK, and C-AT contributed to data collection, measurements, and analysis. CR, JW, and ME contributed to the conceptualization, literature review, and design of the study. CB, CR, JW, and C-AT contributed to the formal analysis and edited the manuscript. All authors agreed to be accountable for all aspects of work ensuring integrity and accuracy, and have reviewed and approved the submitted manuscript for publication.

## Footnotes

1
https://www.slicer.org/

2
https://github.com/Deep-MI/FastSurfer/commit/dabf1e02e6253cac8bd3d641958b01e5348ea0e7

3
https://fsl.fmrib.ox.ac.uk/fsl/fslwiki/
