# Supplementary material for: Metric magnetic resonance imaging analysis reveals pronounced substantia-innominata atrophy in dementia with Lewy bodies with a psychiatric onset
Source: Front Aging Neurosci. 2022 Oct 5;14:815813. doi: 10.3389/fnagi.2022.815813 (PMC9580213; doi:10.3389/fnagi.2022.815813)
Supplement: Supplementary file 1 [file Table_1.pdf]

Supplemental Table 1A: Multiple variables t-Test for group MCI vs. PSY

		MCI		PSY		T-test
	**Parameter**	**Mean**	**SD**	**Mean**	**SD**	**P value**
Basic parameters	AGE (years)	74.7	6.58	76.0	7.40	0.486268
	T1 MPRAGE (1-yes, 0-no)	0.6	0.50	0.8	0.38	**0.045773**
	LiqPos05 (1-yes, 0-no)	0.8	0.44	0.9	0.34	0.262919
	LiqPos06 (1-yes, 0-no)	0.7	0.48	0.7	0.46	0.821018
	SI distance left (mm)	0.67	0.13	0.60	0.11	**0.017944**
	SI distance right (mm)	0.58	0.09	0.59	0.09	0.700832
Brain basic and white matter volumes (mm^3)	Left-Cerebral-White-Matter	235182	39474	234893	40965	0.977903
	Left-Lateral-Ventricle	28561	11712	26057	12579	0.428022
	Left-Inf-Lat-Vent	1609	821	1624	1028	0.950875
	Left-Cerebellum-White-Matter	13384	2376	14127	1978	0.193276
	Left-Cerebellum-Cortex	51852	7821	51357	6089	0.785332
	Left-Thalamus	5942	1100	5819	805	0.623865
	Left-Caudate	3247	913	3176	639	0.731641
	Left-Putamen	3793	1069	3907	694	0.626855
	Left-Pallidum	1812	454	1815	268	0.969042
	3rd-Ventricle	2317	695	2253	599	0.702775
	4th-Ventricle	1986	636	1947	621	0.814942
	Brain-Stem	20177	2890	20238	2263	0.927903
	Left-Hippocampus	3312	541	3237	426	0.552149
	Left-Amygdala	1302	346	1334	312	0.708900
	CSF	1601	492	1587	354	0.897825
	Left-Accumbens-area	1367	5981	1796	8049	0.815592
	Left-VentralDC	3872	552	3819	522	0.702270
	Left-choroid-plexus	1060	304	1115	199	0.417237
	Right-Cerebral-White-Matter	232239	38149	234561	39449	0.817577
	Right-Lateral-Ventricle	27444	10956	23877	11106	0.215538
	Right-Inf-Lat-Vent	1716	918	1502	784	0.335666
	Right-Cerebellum-White-Matter	13152	2923	13679	1973	0.416626
	Right-Cerebellum-Cortex	52012	7230	52091	5607	0.962517
	Right-Thalamus	5826	1184	5718	903	0.693825
	Right-Caudate	3060	876	3055	672	0.982854
	Right-Putamen	3755	1099	3856	651	0.667478
	Right-Pallidum	1808	504	1791	315	0.878817
	Right-Hippocampus	3340	535	3298	484	0.747225
	Right-Amygdala	1450	425	1464	322	0.891548
	Right-Accumbens-area	310	133	371	126	0.072770
	Right-VentralDC	3914	546	3782	559	0.359337
	Right-choroid-plexus	1117	345	1133	212	0.837582

	WM-hypointensities	6205	4424	5348	5111	0.489942
	CC_Posterior	896	242	950	172	0.324843
	CC_Mid_Posterior	554	167	528	143	0.516303
	CC_Central	693	240	693	198	0.997718
	CC_Mid_Anterior	613	304	607	271	0.937834
	CC_Anterior	930	233	945	203	0.796915
Cortex volumes (mm³)	ctx-lh-caudalanteriorcingulate	2277	606	2376	394	0.454429
	ctx-lh-caudalmiddlefrontal	4571	1406	4720	1537	0.695354
	ctx-lh-cuneus	2774	479	2719	402	0.630334
	ctx-lh-entorhinal	1662	569	1866	479	0.137650
	ctx-lh-fusiform	5710	1354	6048	1269	0.322198
	ctx-lh-inferiorparietal	8774	1562	9297	1998	0.263817
	ctx-lh-inferiortemporal	9689	1891	10159	1678	0.313418
	ctx-lh-isthmuscingulate	1959	422	2012	408	0.621898
	ctx-lh-lateraloccipital	10292	1657	10406	1654	0.789890
	ctx-lh-lateralorbitofrontal	6724	1233	6790	817	0.807755
	ctx-lh-lingual	4678	989	4771	960	0.713397
	ctx-lh-medialorbitofrontal	3572	749	3589	492	0.920796
	ctx-lh-middletemporal	10729	2255	10928	1698	0.699825
	ctx-lh-parahippocampal	1593	396	1697	343	0.279348
	ctx-lh-paracentral	2984	576	3363	672	**0.022395**
	ctx-lh-parsopercularis	3131	699	3048	578	0.621697
	ctx-lh-parsorbitalis	1394	390	1587	348	**0.048452**
	ctx-lh-parstriangularis	3281	711	3164	641	0.506173
	ctx-lh-pericalcarine	1419	363	1323	346	0.295677
	ctx-lh-postcentral	7778	1155	8098	1562	0.369959
	ctx-lh-posteriorcingulate	2648	452	2573	428	0.510392
	ctx-lh-precentral	9992	1765	10415	1695	0.347594
	ctx-lh-precuneus	6436	1096	6793	1044	0.202609
	ctx-lh-rostralanteriorcingulate	2769	707	2851	441	0.591766
	ctx-lh-rostralmiddlefrontal	8256	1715	8463	1502	0.621862
	ctx-lh-superiorfrontal	17693	3453	17897	3585	0.822879
	ctx-lh-superiorparietal	7532	1515	7495	1386	0.923402
	ctx-lh-superiortemporal	12861	2593	13116	1956	0.667846
	ctx-lh-supramarginal	7360	1368	7667	1407	0.394301
	ctx-lh-transversetemporal	761	193	748	188	0.805194
	ctx-lh-insula	4755	1013	4955	907	0.423963
	ctx-rh-caudalanteriorcingulate	1629	541	1733	359	0.385588
	ctx-rh-caudalmiddlefrontal	4251	1306	4069	1146	0.568143
	ctx-rh-cuneus	2450	556	2575	507	0.367700
	ctx-rh-entorhinal	1714	488	1811	506	0.451089
	ctx-rh-fusiform	5673	1523	6205	1151	0.132496
	ctx-rh-inferiorparietal	9680	2299	10247	1609	0.272866
	ctx-rh-inferiortemporal	9530	2187	10220	1702	0.178302

	ctx-rh-isthmuscingulate	1886	414	1840	337	0.638252
	ctx-rh-lateraloccipital	10195	1932	10345	1748	0.753122
	ctx-rh-lateralorbitofrontal	7226	1241	7116	849	0.691154
	ctx-rh-lingual	4763	869	4912	903	0.516580
	ctx-rh-medialorbitofrontal	3462	595	3540	430	0.562587
	ctx-rh-middletemporal	10709	2494	10938	1782	0.683567
	ctx-rh-parahippocampal	1492	357	1580	281	0.295454
	ctx-rh-paracentral	2917	564	3309	645	**0.014880**
	ctx-rh-parsopercularis	3036	672	2953	599	0.616380
	ctx-rh-parsorbitalis	1564	357	1595	318	0.727353
	ctx-rh-parstriangularis	3057	782	2795	658	0.165898
	ctx-rh-pericalcarine	1581	381	1483	343	0.303069
	ctx-rh-postcentral	7258	1428	7785	1532	0.172858
	ctx-rh-posteriorcingulate	2550	535	2733	420	0.145339
	ctx-rh-precentral	9822	1845	10053	1586	0.604911
	ctx-rh-precuneus	6680	1206	7067	1059	0.191402
	ctx-rh-rostralanteriorcingulate	1976	532	2072	399	0.431348
	ctx-rh-rostralmiddlefrontal	8219	1891	8449	1810	0.632682
	ctx-rh-superiorfrontal	19351	3625	19915	3845	0.561339
	ctx-rh-superiorparietal	7020	1722	7583	1794	0.219788
	ctx-rh-superiortemporal	12600	2527	12767	1884	0.772543
	ctx-rh-supramarginal	6401	1501	6801	1319	0.277640
	ctx-rh-transversetemporal	529	149	583	139	0.157262
	ctx-rh-insula	4949	976	5036	719	0.697200

Supplemental Table 1A - Legend: Beta-Amyloid-Ratio of the cerebral spinal fluid > 0.5, respectively 0.6, CC – corpus callosum, CSF – cerebrospinal fluid, ctx – cortex, DC – diencephalon, lh - left hemisphere, LiqPos05/06 (1-yes, 0-no), MCI = mild cognitive impairment onset, PSY = psychiatric onset, rh - right hemisphere, SD = standard deviation, WM –white matter.

Table 1B: Multiple variables t-Test for group MCI vs. PSY (T1 MPRAGE only)

		**MCI**		**PSY**		**T-test**
	**Parameter**	**Mean**	**SD**	**Mean**	**SD**	**P value**
Basic parameters	AGE (years)	76.9	6.36	76.1	7.67	0.739025
	LiqPos05 (1-yes, 0-no)	0.7	0.47	0.9	0.37	0.301715
	LiqPos06 (1-yes, 0-no)	0.6	0.51	0.7	0.49	0.709011
	SI distance left (mm)	**0.69**	**0.14**	**0.59**	**0.11**	**0.009154**
	SI distance right (mm)	0.59	0.07	0.60	0.09	0.739736
Brain basic and white matter volumes (mm^3)	Left-Cerebral-White-Matter	226378	35200	227378	40179	0.932947
	Left-Lateral-Ventricle	27814	9840	25894	12559	0.592404
	Left-Inf-Lat-Vent	1610	772	1609	1018	0.996842
	Left-Cerebellum-White-Matter	13519	1726	13856	1979	0.564424
	Left-Cerebellum-Cortex	50644	6926	50493	5900	0.939113
	Left-Thalamus	5663	777	5784	857	0.636699
	Left-Caudate	3266	807	3270	585	0.985465
	Left-Putamen	3969	932	4024	679	0.825039

	Left-Pallidum	1825	408	1834	269	0.930001
	3rd-Ventricle	2280	587	2207	641	0.706890
	4th-Ventricle	1977	692	1937	674	0.852305
	Brain-Stem	20023	2411	20150	2454	0.867080
	Left-Hippocampus	3273	519	3253	459	0.896106
	Left-Amygdala	1330	341	1296	292	0.727083
	CSF	1654	580	1495	278	0.237769
	Left-Accumbens-area	2112	7716	2097	8815	0.995582
	Left-VentralDC	3840	427	3812	566	0.862419
	Left-choroid-plexus	1128	208	1087	169	0.482445
	Right-Cerebral-White-Matter	224758	34037	227941	39103	0.782661
	Right-Lateral-Ventricle	25645	7534	23831	10495	0.534940
	Right-Inf-Lat-Vent	1780	923	1486	774	0.264484
	Right-Cerebellum-White-Matter	13582	2529	13474	1746	0.868663
	Right-Cerebellum-Cortex	51988	6887	51357	5680	0.744173
	Right-Thalamus	5552	858	5707	944	0.582488
	Right-Caudate	3159	723	3191	619	0.877054
	Right-Putamen	3877	874	3940	657	0.786767
	Right-Pallidum	1822	428	1764	317	0.613907
	Right-Hippocampus	3332	468	3352	493	0.893742
	Right-Amygdala	1459	399	1446	310	0.903786
	Right-Accumbens-area	323	93	383	123	0.086847
	Right-VentralDC	3900	451	3746	597	0.360760
	Right-choroid-plexus	1265	265	1125	199	0.055788
	WM-hypointensities	6475	4888	4799	3849	0.215545
	CC_Posterior	911	159	952	187	0.460592
	CC_Mid_Posterior	510	130	506	147	0.931801
	CC_Central	640	216	657	195	0.794852
	CC_Mid_Anterior	537	251	541	243	0.958325
	CC_Anterior	911	191	942	218	0.629700
Cortex volumes (mm^3)	ctx-lh-caudalanteriorcingulate	2435	472	2419	394	0.906861
	ctx-lh-caudalmiddlefrontal	4913	1271	5202	1164	0.443714
	ctx-lh-cuneus	2889	397	2783	385	0.383252
	ctx-lh-entorhinal	1782	464	1844	501	0.679351
	ctx-lh-fusiform	5994	899	6150	1362	0.673882
	ctx-lh-inferiorparietal	8943	1228	9616	1920	0.199044
	ctx-lh-inferiortemporal	9812	1479	10235	1782	0.415339
	ctx-lh-isthmuscingulate	2120	328	2057	419	0.598497
	ctx-lh-lateraloccipital	10339	1508	10599	1677	0.603753
	ctx-lh-lateralorbitofrontal	6765	1042	6795	881	0.918802
	ctx-lh-lingual	4910	410	4927	935	0.940365
	ctx-lh-medialorbitofrontal	3450	647	3553	519	0.564042
	ctx-lh-middletemporal	10591	2364	11067	1801	0.457534
	ctx-lh-parahippocampal	1700	324	1738	342	0.720856

	ctx-lh-paracentral	3266	422	3505	624	0.167886
	ctx-lh-parsopercularis	3287	642	3170	525	0.514632
	ctx-lh-parsorbitalis	1480	289	1658	338	0.077692
	ctx-lh-parstriangularis	3192	547	3262	638	0.707724
	ctx-lh-pericalcarine	1459	373	1371	344	0.431912
	ctx-lh-postcentral	7942	951	8438	1459	0.215008
	ctx-lh-posteriorcingulate	2736	403	2575	449	0.232502
	ctx-lh-precentral	10446	1512	10871	1434	0.354101
	ctx-lh-precuneus	6694	794	6955	1050	0.380495
	ctx-lh-rostralanteriorcingulate	2670	559	2815	438	0.345267
	ctx-lh-rostralmiddlefrontal	8450	1436	8780	1373	0.449317
	ctx-lh-superiorfrontal	18586	2760	18918	2923	0.709012
	ctx-lh-superiorparietal	7790	889	7742	1341	0.894689
	ctx-lh-superiortemporal	12813	2039	13372	1939	0.365910
	ctx-lh-supramarginal	7582	1166	7926	1323	0.382156
	ctx-lh-transversetemporal	749	120	772	184	0.648375
	ctx-lh-insula	5055	655	5117	864	0.802271
	ctx-rh-caudalanteriorcingulate	1766	514	1714	383	0.706365
	ctx-rh-caudalmiddlefrontal	4660	1059	4396	849	0.370093
	ctx-rh-cuneus	2503	515	2654	501	0.341271
	ctx-rh-entorhinal	1771	404	1856	468	0.537727
	ctx-rh-fusiform	5726	1122	6207	1172	0.183487
	ctx-rh-inferiorparietal	9808	2153	10424	1491	0.273364
	ctx-rh-inferiortemporal	9397	1819	10386	1756	0.079912
	ctx-rh-isthmuscingulate	1965	438	1870	343	0.427491
	ctx-rh-lateraloccipital	10203	1927	10384	1683	0.744254
	ctx-rh-lateralorbitofrontal	7187	990	7129	904	0.843173
	ctx-rh-lingual	4848	712	4987	904	0.589582
	ctx-rh-medialorbitofrontal	3430	409	3600	421	0.194182
	ctx-rh-middletemporal	10489	2102	11100	1880	0.323476
	ctx-rh-parahippocampal	1587	297	1601	287	0.874830
	ctx-rh-paracentral	3188	386	3411	620	0.185653
	ctx-rh-parsopercularis	3062	407	3053	572	0.955752
	ctx-rh-parsorbitalis	1548	342	1636	327	0.395358
	ctx-rh-parstriangularis	2947	497	2827	701	0.537063
	ctx-rh-pericalcarine	1559	384	1511	339	0.665358
	ctx-rh-postcentral	7444	1111	8027	1481	0.166826
	ctx-rh-posteriorcingulate	2644	548	2745	431	0.500697
	ctx-rh-precentral	10130	1350	10399	1507	0.549897
	ctx-rh-precuneus	6859	979	7186	1076	0.313300
	ctx-rh-rostralanteriorcingulate	1987	469	2056	406	0.612537
	ctx-rh-rostralmiddlefrontal	8403	1480	8853	1661	0.364963
	ctx-rh-superiorfrontal	20199	2748	20835	3480	0.523165
	ctx-rh-superiorparietal	7221	991	7880	1776	0.163311

	ctx-rh-superiortemporal	12480	2329	13010	1787	0.402923
	ctx-rh-supramarginal	6541	1154	7038	1219	0.184692
	ctx-rh-transversetemporal	542	122	600	143	0.169834
	ctx-rh-insula	5158	595	5111	743	0.825521

Supplemental Table 1B - Legend: Beta-Amyloid-Ratio of the cerebral spinal fluid > 0.5, respectively 0.6, CC – corpus callosum, CSF – cerebrospinal fluid, ctx – cortex, DC – diencephalon, lh - left hemisphere, LiqPos05/06 (1-yes, 0-no), MCI = mild cognitive impairment onset, PSY = psychiatric onset, rh - right hemisphere, SD = standard deviation, WM –white matter.
